# Biomarkers of cardio-renal damage in chronic kidney disease: one size cannot fit all

**DOI:** 10.1186/cc13834

**Published:** 2014-04-17

**Authors:** Davide Bolignano, Giuseppe Coppolino

**Affiliations:** 1 CNR – Institute of Clinical Physiology, c/o EUROLINE, Via Vallone Petrara 55-57, 89124 Reggio Calabria, Italy; 2 Nephrology and Dialysis Unit, University Hospital ‘Magna Graecia’,Viale Europa SNC, 88100 Germaneto, Catanzaro, Italy

## Abstract

Biomarkers are useful tools for diagnosis and risk assessment of acute kidney injury and acute heart failure, particularly in ICU patients. Most biomarkers are produced or cleared by the kidney, so the presence of chronic kidney disease may affect their clinical reliability, particularly if the putative diagnosis of acute kidney injury or acute heart failure is based on a single measurement/single threshold approach. Better alternatives, such as establishing different diagnostic cutoff values per different chronic kidney disease strata or evaluating the diagnostic performance of a delta value (change from baseline levels) instead of a single threshold, should be carefully considered in critically ill patients with renal impairment and other co-morbidities.

Chronic kidney disease (CKD) is highly prevalent in the ICU population and conveys a higher risk of developing both acute kidney injury (AKI) and acute heart failure (AHF). Early serum and urine biomarkers of AKI and AHF may improve diagnosis and risk stratification. Most biomarkers are affected by renal function impairment, however, so the presence of CKD may hamper their predictive capacity.

## 

Donadio investigated whether the presence of underlying CKD may affect the diagnostic performance of two of the most studied biomarkers of cardio-renal damage, namely neutrophil gelatinase-associated lipocalin (NGAL) and brain natriuretic peptide (BNP) [1]. In this study, plasma NGAL increased in a parallel fashion with the reduction in glomerular filtration rate (GFR), generating a very high number of false positive diagnoses of AKI in stable CKD patients. Conversely, urinary NGAL and plasma BNP were less affected by reduced GFR.

NGAL is a kidney stress protein, the levels of which increase in plasma and urine of patients developing AKI, 2 to 8 hours after renal injuries of various nature [2]. Although the predictive performance of NGAL was remarkable in pediatric populations, conflicting results emerged in the adult setting. In particular, studies reporting an excellent discriminant ability mostly excluded patients with preoperative CKD, whereas a more modest performance was reported in those also including patients with impaired renal function [3]. In CKD patients, NGAL levels are strictly, independently and inversely correlated to residual GFR, probably as the consequence of an active production by the damaged but still vital tubule that goes along with the functional decrease of renal parenchyma [4].

The effect of baseline renal function on the diagnostic performance of urinary NGAL was also evaluated recently by McIlroy and colleagues in a prospective observational study in 426 adults undergoing major cardiac surgery [5]. In patients with baseline GFR >60 ml/minute who developed AKI, urinary NGAL was higher at all postoperative time points compared with those patients who did not have this complication. Conversely, no differences were reported in patients with baseline GFR <60 ml/minute. Postoperative NGAL best identified AKI in patients with baseline GFR 90 to 120 ml/minute, therefore suggesting that the optimal discriminatory performance is achieved in patients with normal preoperative renal function.

As is well known, BNP is mostly useful for screening and prognosis of acute and chronic heart dysfunction. Although less than 5% of BNP is cleared by the renal route, accruing evidence indicates that the circulating levels of this biomarker are elevated in CKD patients despite an apparently conserved ventricular function [6,7]. In addition, elevated BNP levels predict accelerated progression of CKD in patients with mild to moderate renal impairment, independently from cardiac (dys)function [8]. Although BNP levels could therefore be helpful in the management of combined heart and kidney disease, these observations might raise concerns regarding the specificity of BNP as biomarker of AHF in the scenario of CKD. Challenging the issue of baseline renal function is thus crucial for validating the potential usefulness of biomarkers in clinical practice. Even the measurement of widely implemented biomarkers, such as troponins or tumor markers [9,10], often leads to misleading clinical interpretations in CKD (especially dialysis) patients.

For biomarkers of AKI and AHF, we believe that, as a general rule, one size (that is, one threshold) cannot fit all. In other words, it is virtually impossible to consider univocal cutoff values able to stratify the risk of AKI or AHF in a miscellaneous population of critically ill patients as a whole. Considering different diagnostic thresholds for different CKD strata might help to improve the overall performance of NGAL and BNP but, predictably, this would not reduce the ground noise caused by other confounders. For instance, apart from renal damage, NGAL levels are known to be affected also by proteinuria, systemic inflammation, oxidative stress, iron balance, diabetes and existing cardiovascular disease [11], while BNP can be influenced by chronic heart failure, obesity, inflammation and even age, gender and heart rate [12]. This variation makes it virtually impossible to find baseline values of these biomarkers falling within the normal range, even in ICU patients with normal renal function or early stage CKD. In view of this, a single-time, threshold-based measurement of NGAL or BNP as an exclusive tool for predicting AKI or AHF should probably be called into question. Even if a cost–benefit analysis must always be taken into account, it would be good to verify whether the predictive capacity of a delta value (variation in biomarkers levels between end of procedure and baseline; for example, time of admission in the ICU) would be superior to that of a single biomarker measurement.

In conclusion, the presence of CKD may actually impact upon the diagnostic performance and reliability of biomarkers of acute damage of kidney and heart. This holds true for biomarkers already used in daily clinical practice as well as for those emerging biomarkers whose promising, preliminary findings in homogeneous study cohorts still await an external validation. Since CKD is exceedingly prevalent in ICU patients, taking systematically into account the effect of residual renal function might enhance the diagnostic performance of biomarkers for the risk stratification of cardio-renal complications in critically ill patients.

## Abbreviations

AHF: Acute heart failure; AKI: Acute kidney injury; BNP: Brain natriuretic peptide; CKD: Chronic kidney disease; GFR: Glomerular filtration rate; NGAL: Neutrophil gelatinase-associated lipocalin.

## Competing interests

The authors declare that they have no competing interests.

## References

[B1] Donadio C Effect of glomerular filtration rate impairment on diagnostic performance of neutrophil gelatinase-associated lipocalin and B-type natriuretic peptide as markers of acute cardiac and renal failure in chronic kidney disease patients Crit Care 2014 18 R39 10.1186/cc13752 24581340 PMC4057335

[B2] Di Grande A Giuffrida C Carpinteri G Narbone G Pirrone G Di Mauro A Calandra S Noto P Le Moli C Alongi B Nigro F Neutrophil gelatinase-associated lipocalin: a novel biomarker for the early diagnosis of acute kidney injury in the emergency department Eur Rev Med Pharmacol Sci 2009 13 197 200 19673171

[B3] Haase M Bellomo R Devarajan P Schlattmann P Haase-Fielitz A NGAL Meta-analysis Investigator Group Accuracy of neutrophil gelatinase-associated lipocalin (NGAL) in diagnosis and prognosis in acute kidney injury: a systematic review and meta-analysis Am J Kidney Dis 2009 54 1012 1024 10.1053/j.ajkd.2009.07.020 19850388

[B4] Bolignano D Donato V Coppolino G Campo S Buemi A Lacquaniti A Buemi M Neutrophil gelatinase-associated lipocalin (NGAL) as a marker of kidney damage Am J Kidney Dis 2008 52 595 605 10.1053/j.ajkd.2008.01.020 18725016

[B5] McIlroy DR Wagener G Lee HT Neutrophil gelatinase-associated lipocalin and acute kidney injury after cardiac surgery: the effect of baseline renal function on diagnostic performance Clin J Am Soc Nephrol 2010 5 211 219 10.2215/CJN.04240609 20056755 PMC2827603

[B6] Austin WJ Bhalla V Hernandez-Arce I Isakson SR Beede J Clopton P Maisel AS Fitzgerald RL Correlation and prognostic utility of B-type natriuretic peptide and its amino-terminal fragment in patients with chronic kidney disease Am J Clin Pathol 2006 126 506 512 10.1309/M7AAXA0J1THMNCDF 16938661

[B7] Jafri L Kashif W Tai J Siddiqui I Azam I Shahzad H Ghani F B-type natriuretic peptide versus amino terminal pro-B type natriuretic peptide: selecting the optimal heart failure marker in patients with impaired kidney function BMC Nephrol 2013 14 117 10.1186/1471-2369-14-117 23725445 PMC3680180

[B8] Yasuda K Kimura T Sasaki K Obi Y Iio K Yamato M Rakugi H Isaka Y Hayashi T Plasma B-type natriuretic peptide level predicts kidney prognosis in patients with predialysis chronic kidney disease Nephrol Dial Transplant 2012 27 3885 3891 10.1093/ndt/gfs365 23114906

[B9] Coppolino G Focus on high sensitivity cardiac troponin tests in patients with altered renal function G Ital Nefrol 2011 28 138 21488024

[B10] Coppolino G Bolignano D Rivoli L Presta P Fuiano G Tumour markers and kidney function: a systematic review Biomed Res Int 2014 Epub ahead of print. [http://dx.doi.org/10.1155/2014/647541] 10.1155/2014/647541 PMC3933284 24689048

[B11] Bolignano D Coppolino G Lacquaniti A Buemi M From kidney to cardiovascular diseases: NGAL as a biomarker beyond the confines of nephrology Eur J Clin Invest 2010 40 273 276 10.1111/j.1365-2362.2010.02258.x 20415702

[B12] Clerico A Giannoni A Vittorini S Passino C Thirty years of the heart as an endocrine organ: physiological role and clinical utility of cardiac natriuretic hormones Am J Physiol Heart Circ Physiol 2011 301 H12 H20 10.1152/ajpheart.00226.2011 21551272

